# Working while sick: validation of the multidimensional presenteeism exposures and productivity survey for nurses (MPEPS-N)

**DOI:** 10.1186/s12913-019-4373-x

**Published:** 2019-08-02

**Authors:** Juliana Nga Man Lui, Janice Mary Johnston

**Affiliations:** School of Public Health, Li Ka Shing Faculty of Medicine, The University of Hong Kong, G/F Patrick Manson Building (North Wing), 7 Sassoon Road, Pokfulam, Hong Kong, Special Administrative Region of China

**Keywords:** Human resource management, Presenteeism, Job demands and resources, Work stress, Work engagement, Productivity, Nurse

## Abstract

**Background:**

Presenteeism is the employee behaviour of physically attending work with reduced performance due to illness or for other reasons. Nurses are four times more likely to exhibit presenteeism compared to other occupations, threatening patient safety through increased patient falls, medication errors and staff-to-patient disease transmission. There is a paucity of standardized instruments that quantify the association between presenteeism with its exposures and related productivity. This study aims to validate an instrument that comprehensively measures presenteeism workplace and personal exposures specifically for Asian nurses.

**Methods:**

Questionnaire domain items were selected based on the JD-R framework and a previously conducted systematic review of pre-existing validated scales measuring work attendance exposures used in previous healthcare studies. The preliminary questionnaire consisted of two outcomes (presenteeism frequency, productivity) and five exposure domains: work resources, work demands, work stress, work engagement, personal traits and health.

Content validation and back translation (English-Cantonese Chinese-English) were carried out. Responses from full-time nurses working in two acute care hospitals (Preliminary questionnaire at Hospital 1: *N* = 295 and main round questionnaire at Hospital 2: *N* = 1146) were included in the validation study to ensure an adequate sample size of ten cases per indicator variable for CFA analysis. A random sample of 80 nurses from Hospital 1 were selected for test-retest reliability 4 weeks post the initial survey. Internal consistency, convergent and discriminant validity tests were also tested.

**Results:**

Satisfactory internal consistency (Cronbach’s alpha > 0.7), test-retest reliability (ICC > 0.4); and construct validity - convergent and discriminant validity was achieved. Confirmatory factor analysis yielded satisfactory fitness indices (CFI and TLI > 0.95, RMSEA < 0.08). Presenteeism and productivity significantly associated with all work resources, work engagement and work stress constructs in Hospital 2.

**Conclusion:**

A reliable Multidimensional Presenteeism Exposures and Productivity Survey (MPEPS-N) has been validated in two distinct hospital environments. The instrument helps to identify and quantify organizational or individual exposures that significantly associate with presenteeism and its related productivity, thus allowing hospital managers to set evidence-based intervention targets for wellness programs and formulate human resource policies in reducing presenteeism-related productivity loss.

**Electronic supplementary material:**

The online version of this article (10.1186/s12913-019-4373-x) contains supplementary material, which is available to authorized users.

## Background

To maintain a sustainable workforce in face of increasing employer-borne healthcare costs, management experts in recent years have sought ways to reduce indirect employee healthcare expenses such as absence replacement and related productivity loss costs [1–3]. Earlier employee health productivity costing studies considered only direct healthcare expenses and sickness absence costs [4, 5], however a study reported that indirect presenteeism illness-related productivity loss costs were found to be 11.5 times higher than direct medical costs of taking sick leave [6].

Presenteeism is the employee behaviour of physically attending work with reduced performance due to illness or for other reasons [7]. Productivity is the performance measure of efficiency and effectiveness of employees when they are at work [8], where productivity may be limited by employee health conditions at work such as presenteeism. Nurses are four times more likely to work while sick compared to other health care or social welfare workers [9]; such behavior is shown to impact patient safety through increased patient falls, medication errors and staff-to-patient disease transmission [10, 11]. As nurses constitute the largest proportion of paid healthcare workforce [12], their productivity, healthcare and welfare costs constitute a substantial portion of hospital expenses, healthcare researchers started to gain interest in nurse presenteeism research [10, 13–15].

Available presenteeism instruments measure frequency of sickness presenteeism episodes [9], presenteeism-related productivity loss and related costs (estimated by employee’s salary discounted by self-rated on-the job productivity levels reductions) [16–18], and employee health and related medical costs [1, 19]. However, existing measures focus on estimating the impact of presenteeism (organizational productivity or monetary loss) but not helpful in identifying evidence-based human resources intervention targets to reduce presenteeism. As suggested in our systematic review on presenteeism exposures and outcomes amongst hospital doctors and nurses [20], despite attempts to carry out studies on the association between presenteeism and its work-related exposures by healthcare researchers [13, 21, 22], the heterogeneity, limited quality of selected measurement tools and paucity in theoretical framework adoption in studies restrict systematic investigation and generalizability of findings, limiting further research progress on management intervention targets in reducing nurse presenteeism [20]. Thus, a questionnaire based on a common theoretical framework with standardized valid and reliable measures on psychometric-related presenteeism exposures is needed.

**Fig. 1:**
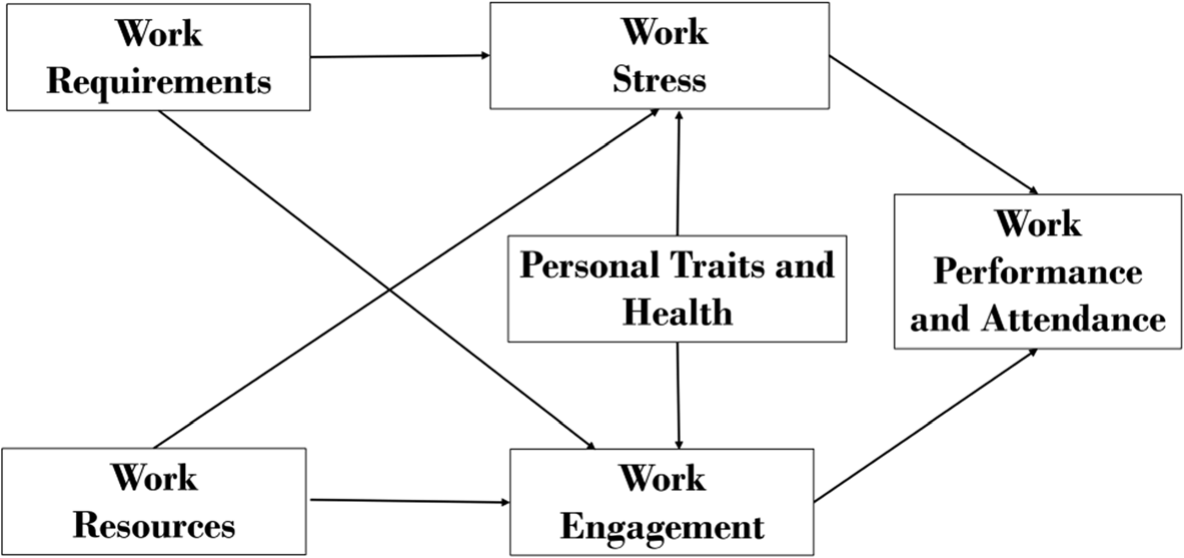
Theoretical framework of presenteeism exposures on employee psychosocial emotions, work performance and attendance

This study is based on the Jobs Demands-Resources (JD-R) model, a popular framework for nurse organizational behavioural studies [20]. JD-R hypothesizes a bidirectional nature /dual process pathway between work resources (e.g., task significance, work schedule, social support, social feedback and organizational care, rewards) and work demands (e.g., physical demands, team psychological demands, effort and ease of substitution) on employee psychosocial emotions (work engagement and work stress), leading to downstream employee outcomes (presenteeism and productivity) [23] (Fig. 1). Other than work-related factors, personal health and traits (e.g. health locus of control) were postulated to impact presenteeism and thus is included in our research framework [2, 24–26].

In Asia, nurses more commonly face acute manpower shortages, high patient to bed ratios and not infrequently large-scale infectious disease outbreaks [27–29]. Asian nurses, perhaps influenced both by work related and societal culture (more collectivist than in the west) are more prone to exhibit presenteeism, which may lead to long term health issues and high turnover rates [30, 31]. More culturally relevant organizational and employee related behavioural research is needed to inform the management and human resources policies and strategies necessary to reach pareto optimality (e.g., allocating limited resources to achieve maximized productivity) is required [31, 32].

This study aims to validate the Multidimensional Presenteeism Exposures and Productivity Survey for Nurses (MPEPS-N) to support the comprehensive measurement of workplace presenteeism and exposures among Asian nurses.

## Methods

### Questionnaire development

**Fig. 2:**
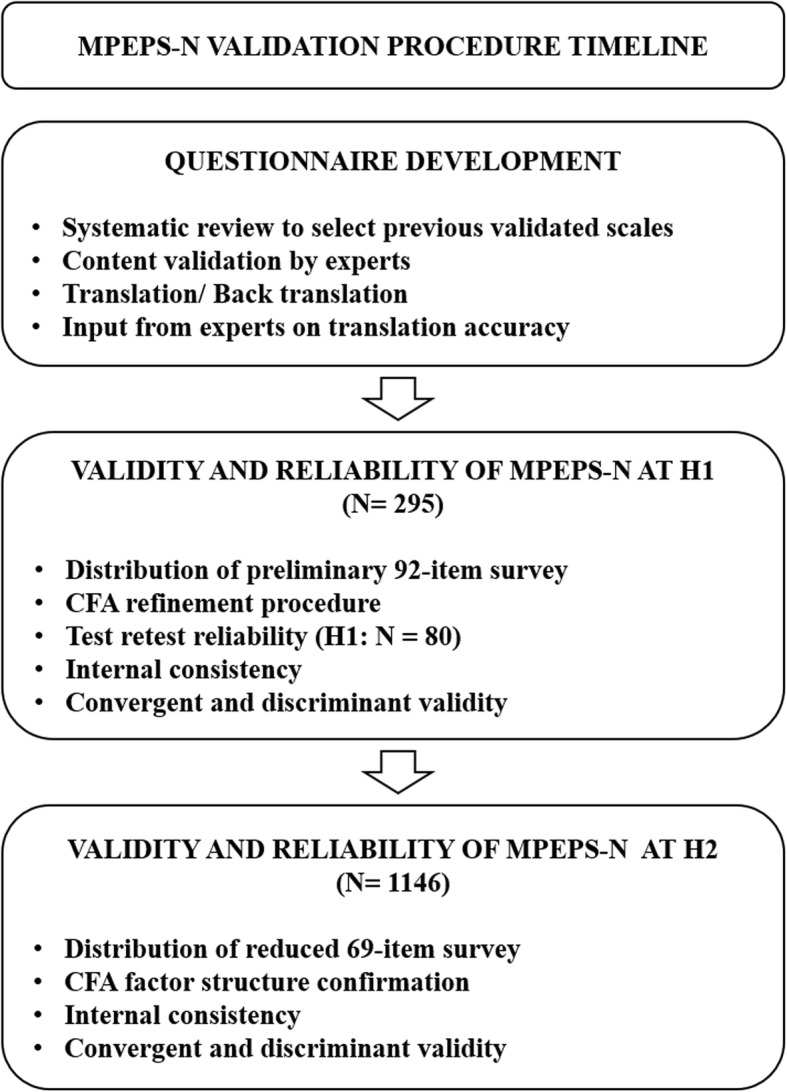
MPEPS-N Validation Procedure Timeline

The MPEPS-N validation procedure timeline is listed in Fig. 2. In the first step, presenteeism exposure domain items, informed by a previously conducted systematic review [20], from which pre-existing validated scales measuring attributes of work attendance previously used in previous JD-R model studies amongst hospital frontline healthcare professionals (nurses and doctors) were extracted.

#### Measures

Some of the selected items [9, 33] or scales in the Multidimensional Presenteeism Exposures and Productivity Survey for Nurses (MPEPS-N) such as work design questionnaire (WDQ) [34], Nordic Psychological & Social Factors at Work (QPS Nordic) [35], Dutch Musculoskeletal Questionnaire [36] do not have validated Chinese versions (Table 1). Whereas Effort-reward imbalance (ERI-S) [37] was translated only in Mandarin Chinese and tested amongst mainland Chinese healthcare workers [38] . The working culture and language (Cantonese Chinese) is distinctly different from our sample of Hong Kong nurses. Maslach burnout Inventory (MBI) [39] was validated and widely used in Hong Kong education sector but not healthcare sector [40], while only the English version was used in a Hong Kong nursing student burnout survey [41]. Utrecht work engagement questionnaire (UWES) has a Cantonese translation but has been tested amongst elderly workers only [42], in which the respondent characteristics are distinctly different from our sample (acute hospital nurses). Multidimensional Health locus of control (MHLC) form C but not Form A has a validated Cantonese Chinese version, Form C is for responders with existing health condition (e.g. patients), while Form A and B measures “general” health locus of control [43, 44]. Thus, a cross-cultural questionnaire validation study is necessary to establish the validity and reliability of these scales for Hong Kong nurse presenteeism and exposures.

**Table 1:** MPEPS-N domains, subdomains and sample items

Domain	Subdomains	Sample items	Adopted Questionnaire	Item # before CFA	Item # after CFA
Work Resources	Task Significance^a^	*The results of my work can significantly impact the lives of patients under my care*	*Work Design Questionnaire (WDQ)*	4	4
	Work Schedule^a^	*My job allows me to independently determine how to complete my tasks.*		3	2
	Social Support^a^	*My job provides me the opportunity to develop close friendships.*		6	5
	Social Feedback^a^	*I receive feedback on my performance from other people in my organization (such as manager and coworkers).*		3	3
	Organizational Care^a^	*The management of my organization are interested in the health and well-being of the personnel.*	*Nordic Psychological & Social Factors at Work (QPSNordic)*	3	2
	Rewards^a^	*Considering all my efforts and achievements, my job promotion prospects are adequate.*	*Effort-reward imbalance (ERI-S)*	7	3
Work Demands	Initiated interdependence^a^	*Unless my job gets done, other jobs cannot be completed.*	*WDQ*	3	0
	Physical demands^a^	*My job requires a great deal of muscular endurance.*		3	3
	Effort^a^	*I have constant time pressure due to a heavy work load.* *Over the past few years, my job has become more and more demanding.*	*Effort-reward imbalance (ERI-S)*	3	3
	Team psychological job demands^a^	*Doctors do not care enough about patients.* *There is conflict between doctors and nurses over therapy.*	*Herschbach 1992*	4	4
	Work conditions^a^	*The wards/ rooms are cramped* *I have/had muscle pain/discomfort in my body due to work*	*Dutch Musculoskeletal Questionnaire*	2	0
	Ease of Substitution^a^	*I would rather go to work while unfit than to find a colleague to substitute for me.* *Being a healthcare worker professional, I would attend my patients first even when I am unfit to go to work.*	*(Arronson + 4 Self Developed Questions)*	6	3
Work Stress	Depersonalization and emotional exhaustion^b^	*Original items of Maslach Burnout Inventory English version cannot be reproduced here due to copyright issues*	*Maslach burnout Inventory (MBI)*	14	8
Work Engagement	–	*At my job, I feel strong and vigorous.* *I feel happy when I am working intensely.*	*Utrecht work engagement questionnaire (UWES)*	9	8
Personal Health and Traits	Health Locus of Control^a^	*If I get sick, it is my own behavior which determines how soon I get well again.* *If I take the right actions, I can stay healthy.*	*Multidimensional Health locus of control A (MHLC-A)*	6	5
Work Productivity	Presenteeism Frequency^c^	*How many times during the last year have you gone to work when you should have been on sick leave due to your health condition?*	*Aronsson 2000*	1	
	Productivity^d^	*Using the same 0-to-10 scale, how would you rate your overall job performance on the days you worked during the past 4 weeks (28 days)?*	*WHO-health and performance questionnaire (WHO-HPQ)*	7	
Additional Items	Quality of life^e^ General health^e^	*How would you rate your quality of life?* *Compared with people of your age, do you consider that your health condition as.*	*WHO- Quality of Life* *(WHO-QOL BREF)*	2	
Demographics		*Please indicate highest degree of nursing training.* *Please indicate the department that you spend the most of your time in.*	*Thematic household survey (THS) and healthcare manpower survey (HMS)*	6	

^a^5-point Likert scale (1- strongly disagree to 5- strongly agree)

^b^7-point Likert scale (0 - never to 6 - every day)

^c^4-point Likert scale (1 - never, 2 - once, 3–2 to 5 times, 4 - > 5 times)

^d^Visual analogue scale (0- worst performance to 10 - top performance)

^e^5-point Likert scale (1- very poor to 5- very good)

The preliminary questionnaire domains, subdomains, sources, number of items and a sample item from each scale are listed in Table 1 (full version of scale is available upon request). As per the hypothesized model, the preliminary questionnaire consisted of five domains (and subdomains): work resources, work demands, work stress, work engagement, personal traits and health. The attitudinal, perceptual, and personal traits items used either a 5-point Likert scale (1- strongly disagree to 5 - strongly agree) or a 7-point scale (0 - never to 6 - every day) [45].

The work productivity items were adapted from the previously validated World Health Organization Health and Work Performance Questionnaire (WHO-HPQ) short form [46]. Items included self-reported number of hours worked in the past 7 days, number of days missed work in the past 28 days, and number of expected working hours per week; and self-assessed job performance 1) compared with others, 2) last year and 3) during past 28 days on a 10-point Likert scale (0- worst performance, 10- top performance). An additional item on self-assessed sickness presenteeism frequency on a 4-point Likert scale (1 - never, 2 - once, 3–2 to 5 times, 4 - > 5 times) was added [2]. Overall quality of life and general health status (two items) adopted from WHOQOL-BREF were also included. The pre-expert content validated questionnaire also contained six individual co-variates (age, sex, staff grade/rank, education, work schedule (shift/regular hours), department) with 110 items in total.

#### Content validation

A panel of seven international and local experts comprising specialists in psychometrics, nursing research, nursing administration and health economics, policy and management assessed the preliminary items for face and content validity and provided structured comments on relevance and comprehensibility.

Using a content validation index (CVI) each expert assessed the per-item face and content validity on a 4-point scale (1- not relevant, 2- somewhat relevant, 3- quite relevant and 4- highly relevant). Items with an average score lower than 0.8 were discarded [47], leaving 92 items in the preliminary questionnaire after expert content validation.

#### Translation

The questionnaire was back-translated (English-Cantonese Chinese-English) and the translation was then confirmed by international and local Cantonese-Chinese speaking nurses, nurse managers and psychometrics experts and moderated by an ‘editor-in-chief’ to achieve consensus.

#### Questionnaire sample

Two acute hospitals in Hong Kong with distinct organizational structures, hospital size, patient characteristics, religious affiliation, and management style were selected to test the applicability of questionnaire in different hospital environments. All full-time nurses working in the two hospitals (Preliminary questionnaire at Hospital 1: *N* = 295 and reduced main round questionnaire at Hospital 2: *N* = 1146) were invited to participate in the validation study. The selected sample size was adequate as a sample size of ten cases per item is recommended for each CFA analysis [48]. Part-time and outsourced nurses were excluded as they have different work patterns and thus organizational stressors than full-time nurses [49].

A random sample of 80 nurses from Hospital 1 were selected for test-retest reliability 4 weeks post the initial survey. The test-retest reliability sample size was determined using R software package “pwr”, assuming an alpha of 0.05, power of 0.8 to detect a medium effect size of 0.4 and an estimated dropout rate of 60%.

### Model fitness and refinement

Confirmatory factor analysis (CFA) using maximum likelihood (ML) estimation and pairwise deletion of missing values was used to establish scale factor structures [50]. CFA reduction for each presenteeism exposure domain was first carried out in H1 sample, whereas a second round of CFA on the reduced domains were carried out in H2 to confirm the factor structures in different hospital environments (Fig. 2). In each CFA model, items were loaded onto respective subdomains: work resources (6-factor), work demands (6-factor), work stress (2-factor), work engagement (1-factor), health locus of control (1-factor).

Although CFA could not be carried out on a single item (presenteeism) or numerical responses (productivity), self-reported sick leave at H1 was compared with administrative payroll records on the number of sick leaves taken and there was strong correlation (0.93). This method was also used for validation of the WHO-HPQ Persian version in Iranian healthcare workers with comparable correlation between self-reported data and administrative data [51].

To improve model fitness, items with factor loading < 0.4 and standardized residual covariance > 1.96 or < − 1.96 (*p* <  0.05) were deleted. Between error variance paths were added if modification indices (MI) were more than six and were supported by theory or prior research [52].

#### Fit indices

Model fit was evaluated with chi-squared test (χ2/df), and root mean square error of approximation (RMSEA). Relative fit including comparative fit index (CFI), Tucker Lewis index (TLI), Goodness-of-fit index (GFI) and standardized root-mean-square residual (SRMR) were also used. Values of 1) χ2/df ratio < 3.0, 2) CFI, TLI and GFI > 0.95, 3) SRMR and RMSEA < 0.08 were used to assess model fit [53].

### Questionnaire validity and reliability

#### Test-retest reliability

Intra-class correlation coefficient (ICC) 2-way random measurement was used to determine test-retest reliability of the five domains; where ICC values between 0.4 and 0.74, and values greater than 0.75 indicate moderate and excellent reliability respectively [54].

#### Internal consistency

Cronbach’s alpha coefficient was used to assess internal consistency of the domains and subdomains, where alpha values above 0.7 were considered satisfactory [48].

#### Convergent and discriminant validity

Pearson correlation coefficients were used to assess convergent and discriminant validity between domain and subdomain mean scores. Convergent validity was hypothesized to be supported if both pairs of mean domain scores of 1) work resources and work engagement, and 2) the work demands, and work stress are positively correlated. Divergent validity was hypothesized to be supported if the work stress and work engagement mean domain scores are inversely correlated. Health locus of control domain was predicted to be negatively correlated to work stress but positively correlated to work engagement domain.

Presenteeism was hypothesized to be positively associated with work demands and work stress, while negatively associated with work resources, health locus of control, work engagement, quality of life, general health; and vice versa for the hypothesized association between the domains with productivity.

For convergent and divergent validity assessment thresholds, Pearson correlation coefficients of < 0.49, 0.50–0.74 and > 0.75 indicates weak, moderate and strong relationship respectively [54]. For discriminant validity assessment, inter-domain Pearson correlation coefficients are hypothesized to be lower than intra-domain coefficients.

Confirmatory factor analysis (CFA) was performed in R (version 3.4.1) using the “lavaan” package (version 0.5–23.1097). Cronbach’s alpha, ICC values and Pearson correlation coefficients were calculated using SPSS version 24.

### Ethics approval

Ethics approval was obtained from the Institutional Review Board of the University of Hong Kong/Hospital Authority Hong Kong West Cluster (HKU/HA HKW IRB) (reference number: UW 16–102) and Hospital Authority Kowloon West Cluster Research Ethics Committee (reference number: KW/EX-17-028(108–07)).

## Results

### Response rate (RR)

The preliminary 92-item questionnaire was completed by 246 of 295 nurses in H1 (RR_Hospital 1_ = 83%) and main round CFA reduced 69-item questionnaire was completed by 824 of 1146 nurses in H2 (RR_Hospital 2_ = 71.9%). The nurses in Hospital 1 vs Hospital 2 were significantly older, fewer had tertiary level education, were more likely to work day-time shift and fewer were in middle management grades (Table 2). Completed 4-week test-retest questionnaires were returned by 50 out of the 80 (RR = 62.5%) randomly selected nurses at Hospital 1.

**Table 2:** A comparison of demographic and work-related characteristics for nurses in Hospital 1 and 2

Characteristics	Preliminary questionnaire Hospital 1 (*n* = 246) n (%)	Main round Questionnaire Hospital 2 (*n* = 824) n (%)	*P*-value
Gender			
Male	25 (10.2)	95 (11.5)	0.63
Female	221 (89.8)	729 (88.5)	
Age Group			
≤ 30	38 (19.9)	226 (28.4)	< 0.001^**^
31–40	55 (28.8)	196 (24.6)	
41–50	48 (25.1)	263 (33.0)	
≥ 51	50 (26.2)	111 (14.0)	
Education			
Certificate/ Diploma	59 (28.0)	72 (9.1)	< 0.001^**^
Associate Diploma/ Higher Diploma	43 (20.5)	47 (6.0)	
Bachelor’s Degree (BscN/BN)	65 (31.0)	432 (54.8)	
Postgraduate Degree	43 (20.5)	237 (30.1)	
Nurse Ranking			
Junior staff (EN/RN)^a^	198 (80.5)	618 (75.0)	< 0.001^**^
Middle management (APN/NC)	31 (12.6)	171 (20.8)	
Senior management (WM/UM/DOM)	17 (6.9)	35 (4.2)	
Working Schedule			
Shift schedule	164 (69.5)	664 (82.8)	< 0.001^**^
Regular schedule (9 am-6 pm)	72 (30.5)	138 (17.2)	
Department			
A&E and Outpatients^b^	14 (5.9)	94 (12.0)	0.04^*^
Medicine^c^	105 (44.3)	309 (39.6)	
Surgery^d^	90 (38.0)	303 (38.8)	
Others^e^	28 (11.8)	75 (9.6)	

Note: ^a^EN - enrolled nurses, RN- registered nurses, APN – advanced practitioner nurses, WM – ward manager, UM - unit manager, DOM- department operations manager

^b^A&E and outpatients - includes accident and emergency, ambulatory Care and outpatients

^c^Medicine - includes medicine, geriatrics, pediatrics and intensive care unit (ICU)

^d^Surgery - includes surgery, obstetrics, gynecology and operation theatre

^e^0thers - includes administration, management, residential care, public health, rehabilitation, occupational Health, community nursing, mental health, psychiatry, addiction treatment and others

^*^p < 0.05; ^**^p < 0.001

### Confirmatory factor analysis

Confirmatory Factor Analysis for each of Hospital 1 and Hospital 2 domains were independently tested. After carrying out model refinement procedures by deleting items with loadings < 0.4 and standardized residual covariances > 1.96 or < − 1.96 (number of deleted items per subdomain listed in Table 1), satisfactory fit indices were demonstrated for both Hospital 1 and Hospital 2 with values greater than 0.95 (for CFI and TLI) [55]. However fit indices for work engagement (CFI = 0.939, TLI = 0.899) and health locus of control (TLI =0.939) in Hospital 2 were weaker. The items for the subdomains - work conditions and initial independence failed to load on their respective latent constructs, thus the two subdomains were deleted from work demands domain for the CFA analysis. RMSEA for all scales were below 0.08, SRMR values below 0.06, indicating acceptable model fit.

### Internal consistency

Despite differences in some of the domains and subdomains mean scores between Hospitals 1 and 2, Cronbach alpha for all domains (work resources, work demands, work stress, work engagement and health locus of control) and subdomains (task significance, work schedule, social support, social feedback, organizational care, rewards, physical demands, team psych demands, effort, ease of substitution, emotional exhaustion and depersonalization) were satisfactory (domains range: 0.75 to 0.90; subdomains range: 0.65 to 0.96), except for ease of substitution (α = 0.65) (Table 3).

**Table 3:** Means, standard deviations and Cronbach’s alpha coefficient for questionnaire domains and subdomains in Hospitals 1 and 2

	Hospital 1	Hospital 2			
	Alpha	Mean (SD)	Alpha	Mean (SD)	*P*-value
Work Resources	0.90	3.32 (0.54)	0.90	3.36 (0.52)	0.40
Task Significance ^a^	0.90	3.73 (0.74)	0.86	3.75 (0.68)	0.19
Work Schedule ^a^	0.90	3.28 (0.90)	0.87	3.34 (0.87)	0.29
Social Support ^a^	0.77	3.24 (0.69)	0.76	3.36 (0.63)	< 0.001^**^
Social Feedback^a^	0.87	3.39 (0.75)	0.89	3.33 (0.73)	0.24
Organizational Care^a^	0.94	2.61 (0.90)	0.91	2.65 (0.96)	0.57
Rewards^a^	0.75	3.02 (0.74)	0.81	3.16 (0.77)	0.01^*^
Work Demands	0.78	3.23 (0.44)	0.78	3.38 (0.39)	< 0.001^**^
Physical Demands^a^	0.96	3.53 (1.05)	0.93	3.89 (0.83)	< 0.001^**^
Team Psych Demands^a^	0.85	2.76 (0.73)	0.82	2.96 (0.71)	< 0.001^**^
Effort^a^	0.82	4.01 (0.77)	0.80	3.96 (0.72)	0.36
Ease of Substitution^a^	0.65	2.99 (0.92)	0.69	3.10 (0.89)	0.09
Work Engagement^a^	0.87	3.03 (0.74)	0.90	3.09 (0.71)	0.27
Health Locus of Control^a^	0.75	3.13 (0.58)	0.76	2.91 (0.69)	0.06
Work Stress	0.88	2.56 (1.31)	0.89	2.05 (1.31)	< 0.001^**^
Emotional Exhaustion ^b^	0.92	3.24 (1.57)	0.92	2.66 (1.59)	< 0.001^**^
Depersonalization ^b^	0.84	1.42 (1.46)	0.90	1.03 (1.38)	< 0.001^**^

Note: ^a^5-point Likert scale (1- strongly disagree to 5- strongly agree)

^b^7-point Likert scale (0 - never to 6 - every day)

^*^p < 0.05; ^**^p < 0.001

### Convergent and discriminant validity

Inter-domain mean scores were more positively correlated to each other as compared to intra-domain subdomain mean scores, thereby satisfying the convergent/discriminant validity criteria (Additional file 1: Tables S1a and S1b).

Although the ‘ease of substitution’ subdomain mean score both positively correlated with “work engagement” (*r* = 0.30, *p* <  0.01) and “work demands” (*r* = 0.54, *p* < 0.01) in Hospital 1, ‘ease of substitution’ satisfied the convergent and discriminant validity criteria in Hospital 2. Health locus of control (HLOC) was positively correlated with the work demands domain in Hospital 1(*r* = 0.20, *p* < 0.01) but not in Hospital 2.

Hospital 2 data showed significant associations between sickness presenteeism frequency and all psychosocial work domains as hypothesized (Table 4). Fewer work psychosocial domains were significantly associated with the presenteeism measures in Hospital 1 as compared to Hospital 2. Presenteeism and productivity were significantly associated with all work resources, work engagement and work stress constructs in Hospital 2. Although presenteeism significantly correlated with all work demands domains and subdomains, productivity only significantly correlated with team psychological demands within the work demands construct. Health locus of control was significantly negatively associated with productivity but not presenteeism in Hospital 2.

**Table 4:** Correlations between presenteeism productivity and frequency with work psychosocial domains and subdomains in Hospitals 1 and 2

	Hospital 1		Hospital 2	
	Presenteeism	Productivity	Presenteeism	Productivity
Work Resources	0.00	0.25^**^	−0.24^**^	0.21^**^
Task Significance	0.10	0.13^*^	−0.08^*^	0.22^**^
Work Schedule	−0.02	0.21^**^	−0.20^**^	0.10^**^
Social Support	0.04	0.28^**^	− 0.12^**^	0.17^**^
Feedback	0.04	0.22^**^	−0.16^**^	0.18^**^
Organizational Care	−0.12	0.08	−0.26^**^	0.12^**^
Rewards	0.02	0.11	−0.23^**^	0.21^**^
Work Demands	0.28^**^	0.21^**^	0.22^**^	0.01
Physical Demands	0.11	0.08	0.16^**^	−0.06
Team Psych Demands	0.08	0.11	0.14^**^	−0.07^*^
Effort	0.27^**^	0.16^*^	0.23^**^	−0.03
Ease of Substitution	0.23^**^	0.09	0.23^**^	−0.04
Work Engagement	0.03	0.29^**^	−0.12^**^	0.34^**^
Health Locus of Control	−0.01	0.13^*^	−0.13^**^	0.06
Work Stress	0.19^**^	−0.12	0.21^**^	−0.30^**^
Depersonalization	0.10	−0.17^**^	0.08^*^	−0.29^**^
Emotional Exhaustion	0.22^**^	−0.03	0.27^**^	−0.23^**^
Quality of Life	−0.18^**^	0.27^**^	−0.20^**^	0.29^**^
General Health	−0.21^**^	0.27^**^	−0.31^**^	0.23^**^

^*^p < 0.05; ^**^ *p* < 0.001

### Test-retest reliability

The Hospital 1 4-week test-retest ICC domain scores achieved moderate reliability (0.4–0.7), ranging from 0.42 (95% CI: 0.16–0.64) for work stress domain to 0.71 (95% CI: 0.54–0.82) for work resources domain (Table 5). The nurse demographics and characteristics did not differ significantly between those who responded in the test and retest surveys.

**Table 5:** Four-week Test-retest mean scores and intra-class correlation coefficients for the preliminary questionnaire at Hospital 1 (*N* = 50)

Domains	Test	Retest	
	Mean (SD)	Mean (SD)	ICC ^c^ (95% CI)
Work Demands ^a^	3.34 (0.48)	3.22 (0.59)	0.58 (0.37–0.74)
Work Resources ^a^	3.33 (0.57)	3.36 (0.56)	0.71 (0.54–0.82)
Work Stress ^b^	2.95 (1.19)	2.92 (0.81)	0.42 (0.16–0.64)
Work Engagement ^a^	3.26 (0.68)	3.29 (0.58)	0.70 (0.52–0.82)
Personal Health locus of control ^a^	3.04 (0.66)	3.03 (0.64)	0.53 (0.30–0.70)

^a^5-point Likert scale (1- strongly disagree to 5- strongly agree)

^b^7-point Likert scale (0 - never to 6 - every day)

^c^0.4 < ICC < 0.7 confirms that moderate reliability is achieved

## Discussion

As presented in this paper, a reliable presenteeism exposure and productivity questionnaire is developed and validated amongst nurses working in two hospitals. These hospitals have different working environments vis organizational structure, work place demands, patient characteristics, management styles, hospital size and culture. To our knowledge, this scale is the first to measure the association between presenteeism productivity and organizational factors, work place exposures and personal characteristics in nurses working in a densely populated Asian city and serving a rapidly aging post-war baby boomer population.

Although the internal consistency of the ‘ease of substitution’ subdomain (Hospital 1: α =0.65, Hospital 2; α = 0.69) is moderate (α is acceptable at 0.5 and above) as recommended by Nunnally [48], there is strong empirical evidence in this field that the ‘ease of substitution’ subdomain captures a crucial construct that contributes to presenteeism behaviour, therefore a decision was made to retain this subdomain in the instrument [2, 9, 24, 56–58].

The items within the ‘work conditions’ and ‘initial independence’ subdomains failed to load onto the construct as hypothesized, perhaps due to the differences in cultural and work autonomy in the East vs the West. The overall convergent and divergent validity were satisfactory except for the ‘ease of substitution’ subdomain. Attitudes towards ‘ease of substitution’ among nurses working in an East vs West setting may reflect cultural differences related to flexible work schedules and work life balance. Nurse managers in Korea and Japan, though acknowledging the association between work schedule flexibility, job satisfaction and turnover rate, face a dilemma managing the perceived inequity among staff when accommodating request for flexible working schedules assignments [59, 60]. This contrasts with outcomes of a multi-country European study of RNs working in units with higher scheduling flexibility having lower intention to leave [61]. This study indicates the importance of cross-cultural validation as distinct population samples may comprehend or perceive given constructs differently. In this case, a potential evidence-based human resources strategic target (increasing work autonomy and flexibility of working schedule) may be effective in European but not Asian nurses. Researchers are also encouraged to validate the MPEPS scale and test its applicability in other cultural and occupational settings in the future.

Confirmatory factor analysis of the refined survey domains demonstrated satisfactory model fit. The chi-square test result differences as noted between the two hospitals were likely due the differences between sample size, the hospital operational environments (long term care vs district level acute care) and organizational culture particularly of that within the nursing structures.

The inconsistent finding between Hospital 1 vs Hospital 2 in the relationship between WHO-HPQ scale presenteeism score and work psychosocial domains may be explained by a lack of statistical power to detect a difference in Hospital 1 and suggests the need for further work in this area.

Convergent and discriminant validity was found for sickness presenteeism and presenteeism score (WHO-HPQ scale) for all exposures except health locus of control and work demands for Hospital 2 indicating work demands although having an impact on sickness presenteeism frequency, may not play a role in on the job productivity.

Researchers can utilize the validated MPEPS-N and extend existing presenteeism research on employee health-related presenteeism medical and productivity cost burden to evidence-based identification of modifiable cultural and occupational specific human resources intervention targets [1]. Moreover, as few existing studies have established causal relationship between presenteeism and its risk factors [20], researchers are encouraged to adopt MPEPS-N in prospective studies in the future. With the validation of this standardized multi-dimensional presenteeism exposures measure, improvements in the quality, comprehensiveness and generalizability of findings of current nurse presenteeism and exposures research are anticipated with the adoption of MPEPS-N [20].

## Limitations

Data-driven refinements to the hypothesized model and subsequent validated questionnaire are only considered preliminary and representative of Hong Kong public hospital nurses. To further improve the generalizability of this measure, further work must be tested amongst other healthcare professionals. The data used for the validation were collected at two times points 1 year apart, from two distinct hospitals, in two different operational modes: hospital utilization was very high and the nurses were under pressure (during the winter flu surge period) and the second when hospital utilization was considered normal and nurses under less pressure. However, the outcomes demonstrate the stability of the factor structure and the underlying constructs being measured.

The limitations of self-reported questionnaire apply to the MPEPS-N survey, such as social desirability bias (providing socially acceptable answers) and acquiescent response bias (unified response to all questions with “yes”). To reduce social desirability bias, confidentiality and anonymity of responses were assured by taking the following precautions: 1) each survey was labelled with unique identification number (UIN) for test-retest assessment to avoid individual identification during data analysis, 2) self-sealed return envelope was provided, and 3) researchers collected the completed questionnaires at each ward to ensure that hospital management or supervisors did not have access to individual level data responses. Acquiescent response bias was tested by intercalating the order of opposite domain items in the survey. The satisfactory convergent and divergent validity between the domains and subdomains eliminated the possibility of acquiescent response bias for our collected data.

## Conclusion

The MPEPS-N is systematically validated in this paper for use in an Asian healthcare organization setting. As with other research, the study has demonstrated the potential workplace impact or moderating effect of the hypothesized domains on nursing staff. The outcome, which focuses on an organizational and well-being approach is supported by the theoretical work of others. Such a validated instrument will give nurse managers in Asia better information to consider the relationships between the attributes of work demands and work resources, work stress and work engagement when making staff work allocation and job demand decisions. Further research is needed to demonstrate the potential work-related, organizational and personal factors in Asian health care settings that are postulated to impact presenteeism.

## Additional file

Additional file 1: **Table S1a.** Hospital 1 Correlation matrix between the 5-factor domains and internal consistency, **Table S1b.** Hospital 2 Correlation matrix between the 5-factor domains and internal consistency. (DOCX 54 kb)

## Data Availability

The data and questionnaire that support the findings of this study are available on reasonable request from the corresponding author.

## Abbreviations

A&E: Accident and emergency
APN: Advanced practitioner nurses
CFA: Confirmatory factor analysis
CFI: Comparative fit index
CVI: Content validation index
DOM: Department operations manager
EN: Enrolled nurses
ERI-S: Effort reward imbalance scale
GFI: Goodness-of-fit index
ICC: Intraclass correlation coefficient
ICU: Intensive care unit
JD-R: Job-Demands Resources Model
MHLC: Multidimensional Health locus of control
ML: Maximum likelihood
MPEPS-N: Multidimensional Presenteeism Exposures and Productivity Survey (Nurses)
QPS Nordic: Nordic Psychological & Social Factors at Work
RMSEA: Root mean square error of approximation
RN: Registered nurses
RR: Response rate
SRMR: Standardized root-mean-square residual
TLI: Tucker-Lewis index
UM: Unit manager
UWES: Utrecht work engagement scale
WDQ: Work design questionnaire;
WHO-HPQ: World Health Organization Health and Work Performance Questionnaire
WHOQOL-BREF: World Health Organization Quality of Life Instrument, Short Form
WM: Ward manager
