# Supplementary material for: Working while sick: validation of the multidimensional presenteeism exposures and productivity survey for nurses (MPEPS-N)
Source: BMC Health Serv Res. 2019 Aug 2;19:542. doi: 10.1186/s12913-019-4373-x (PMC6679450; doi:10.1186/s12913-019-4373-x)
Supplement: Supplementary file 1 — Table S1a. Hospital 1 Correlation matrix between the 5-factor domains and internal consistency, Table S1b. Hospital 2 Correlation matrix between the 5-factor domains and internal consistency. (DOCX 54 kb) [file 12913_2019_4373_MOESM1_ESM.docx]

**Table S1a. Hospital 1 Correlation matrix between the 5 factor domains and Internal Consistency**

|  | Hospital 1 | 1 | 2 | 3 | 4 | 5 | 6 | 7 | 8 | 9 | 10 | 11 | 12 | 13 | 14 | 15 | 16 | 17 | 18 | 19 | 20 | 21 |
| --- | --- | --- | --- | --- | --- | --- | --- | --- | --- | --- | --- | --- | --- | --- | --- | --- | --- | --- | --- | --- | --- | --- |
| 1 | Work Resources | 1 |  |  |  |  |  |  |  |  |  |  |  |  |  |  |  |  |  |  |  |  |
| 2 | Task Significance | 0.57** | 1 |  |  |  |  |  |  |  |  |  |  |  |  |  |  |  |  |  |  |  |
| 3 | Work Schedule | 0.70** | 0.34** | 1 |  |  |  |  |  |  |  |  |  |  |  |  |  |  |  |  |  |  |
| 4 | Social Support | 0.80** | 0.35** | 0.41** | 1 |  |  |  |  |  |  |  |  |  |  |  |  |  |  |  |  |  |
| 5 | Social Feedback | 0.75** | 0.25** | 0.34** | 0.63** | 1 |  |  |  |  |  |  |  |  |  |  |  |  |  |  |  |  |
| 6 | Organizational Care | 0.69** | 0.11 | 0.28** | 0.50** | 0.46** | 1 |  |  |  |  |  |  |  |  |  |  |  |  |  |  |  |
| 7 | Rewards | 0.57** | 0.08 | 0.27** | 0.57** | 0.52** | 0.56** | 1 |  |  |  |  |  |  |  |  |  |  |  |  |  |  |
| 8 | Work Demands | 0.26** | 0.20** | 0.24** | 0.22** | 0.28** | -0.01 | 0.26** | 1 |  |  |  |  |  |  |  |  |  |  |  |  |  |
| 9 | Physical Demands | -0.06 | 0.07 | 0.04 | -0.11 | 0.04 | -0.25** | -0.14* | 0.62** | 1 |  |  |  |  |  |  |  |  |  |  |  |  |
| 10 | Team Psych Demands | -0.02 | -0.02 | 0.09 | -0.02 | 0.00 | -0.11 | -0.09 | 0.54** | 0.28** | 1 |  |  |  |  |  |  |  |  |  |  |  |
| 11 | Effort | 0.07 | 0.21** | 0.08 | 0.07 | 0.1 | -0.16* | -0.07 | 0.61** | 0.24** | 0.24** | 1 |  |  |  |  |  |  |  |  |  |  |
| 12 | Ease of Substitution | 0.17** | 0.17** | 0.16* | 0.14* | 0.12 | 0.03 | 0.10 | 0.54** | 0.02 | 0.03 | 0.20** | 1 |  |  |  |  |  |  |  |  |  |
| 13 | Work Engagement | 0.51** | 0.26** | 0.32** | 0.46** | 0.44** | 0.34** | 0.36** | 0.21** | -0.06 | -0.05 | 0.04 | 0.30** | 1 |  |  |  |  |  |  |  |  |
| 14 | Health Locus of Control | 0.31** | 0.11 | 0.22** | 0.25** | 0.28** | 0.22** | 0.27** | 0.20** | -0.11 | -0.05 | 0.12 | 0.32** | 0.41** | 1 |  |  |  |  |  |  |  |
| 15 | Work Stress | -0.16* | 0.08 | -0.05 | -0.23** | -0.13* | -0.21** | -0.22** | 0.30** | 0.25** | 0.19** | 0.38** | 0.15* | -0.25** | -0.06 | 1 |  |  |  |  |  |  |
| 16 | Emotional Exhaustion | -0.16* | 0.09 | -0.02 | -0.23** | -0.17** | -0.24** | -0.25** | 0.34** | 0.27** | 0.20** | 0.48** | 0.13* | -0.18** | -0.12 | 0.85** | 1 |  |  |  |  |  |
| 17 | Depersonalization | -0.1 | 0.04 | -0.06 | -0.16* | -0.05 | -0.11 | -0.11 | 0.16* | 0.14* | 0.12 | 0.14* | 0.11 | -0.23** | 0.03 | 0.83** | 0.41** | 1 |  |  |  |  |
| 18 | Quality of Life | 0.40** | 0.02 | 0.18** | 0.44** | 0.38** | 0.39** | 0.41** | -0.04 | -0.11 | -0.18** | -0.24** | 0.04 | 0.40** | 0.23** | -0.35* | -0.39** | -0.18** | 1 |  |  |  |
| 19 | General Health | 0.32** | -0.02 | 0.15* | 0.35** | 0.32** | 0.34** | 0.35** | 0.01 | -0.04 | -0.08 | -0.18** | 0.01 | 0.36** | 0.22** | -0.34* | -0.32** | -0.24** | 0.70** | 1 |  |  |
| 20 | Presenteeism | 0 | 0.1 | -0.02 | 0.04 | 0.04 | -0.12 | 0.02 | 0.28** | 0.11 | 0.08 | 0.27** | 0.23** | 0.03 | -0.01 | 0.19** | 0.22** | 0.1 | -0.18** | -0.21** | 1 |  |
| 21 | Productivity | 0.25** | 0.13* | 0.21** | 0.28** | 0.22** | 0.08 | 0.11 | 0.21** | 0.08 | 0.11 | 0.16* | 0.09 | 0.29** | 0.13* | -0.12 | -0.03 | -0.17* | 0.27** | 0.27** | 0.02 | 1 |

Note. ** Correlation is significant at the 0.01 level (2-tailed).

* Correlation is significant at the 0.05 level (2-tailed).

**Table S1b. Hospital 2 Correlation matrix between the 5 factor domains and Internal Consistency**

|  | Hospital 2 | 1 | 2 | 3 | 4 | 5 | 6 | 7 | 8 | 9 | 10 | 11 | 12 | 13 | 14 | 15 | 16 | 17 | 18 | 19 | 20 | 21 |
| --- | --- | --- | --- | --- | --- | --- | --- | --- | --- | --- | --- | --- | --- | --- | --- | --- | --- | --- | --- | --- | --- | --- |
| 1 | Work Resources | 1 |  |  |  |  |  |  |  |  |  |  |  |  |  |  |  |  |  |  |  |  |
| 2 | Task Significance | 0.57** | 1 |  |  |  |  |  |  |  |  |  |  |  |  |  |  |  |  |  |  |  |
| 3 | Work Schedule | 0.73** | 0.31** | 1 |  |  |  |  |  |  |  |  |  |  |  |  |  |  |  |  |  |  |
| 4 | Social Support | 0.71** | 0.33** | 0.43** | 1 |  |  |  |  |  |  |  |  |  |  |  |  |  |  |  |  |  |
| 5 | Social Feedback | 0.76** | 0.32** | 0.40** | 0.46** | 1 |  |  |  |  |  |  |  |  |  |  |  |  |  |  |  |  |
| 6 | Organizational Care | 0.73** | 0.16** | 0.35** | 0.39** | 0.52** | 1 |  |  |  |  |  |  |  |  |  |  |  |  |  |  |  |
| 7 | Rewards | 0.65** | 0.25** | 0.41** | 0.45** | 0.51** | 0.60** | 1 |  |  |  |  |  |  |  |  |  |  |  |  |  |  |
| 8 | Work Demands | 0.02 | 0.16** | 0.00 | 0.09* | 0.02 | -0.13** | 0.15** | 1 |  |  |  |  |  |  |  |  |  |  |  |  |  |
| 9 | Physical Demands | -0.07* | 0.04 | -0.03 | 0.01 | -0.07 | -0.17** | -0.09** | 0.60** | 1 |  |  |  |  |  |  |  |  |  |  |  |  |
| 10 | Team Psych Demands | -0.29** | -0.10** | -0.21** | -0.18** | -0.23** | -0.27** | -0.25** | 0.50** | 0.18** | 1 |  |  |  |  |  |  |  |  |  |  |  |
| 11 | Effort | -0.18** | 0.11** | -0.18** | -0.07* | -0.09* | -0.31** | -0.17** | 0.64** | 0.29** | 0.29** | 1 |  |  |  |  |  |  |  |  |  |  |
| 12 | Ease of Substitution | -0.05 | 0.09** | -0.01 | 0.01 | -0.07 | -0.15** | -0.10** | 0.60** | 0.10** | 0.12** | 0.27** | 1 |  |  |  |  |  |  |  |  |  |
| 13 | Work Engagement | 0.56** | 0.39** | 0.36** | 0.43** | 0.37** | 0.43** | 0.44** | 0.01 | -0.07 | -0.20** | -0.22** | 0.05 | 1 |  |  |  |  |  |  |  |  |
| 14 | Health Locus of Control | 0.36** | 0.21** | 0.27** | 0.27** | 0.26** | 0.25** | 0.22** | 0.05 | -0.14** | -0.08* | -0.10** | 0.17** | 0.38** | 1 |  |  |  |  |  |  |  |
| 15 | Work Stress | -0.28** | -0.07 | -0.16** | -0.22** | -0.21** | -0.30** | -0.30** | 0.34** | 0.18** | 0.28** | 0.42** | 0.27** | -0.35** | -0.04 | 1 |  |  |  |  |  |  |
| 16 | Emotional Exhaustion | -0.29** | -0.02 | -0.18** | -0.23** | -0.21** | -0.35** | -0.32** | 0.36** | 0.21** | 0.26** | 0.46** | 0.29** | -0.34** | -0.08* | 0.87** | 1 |  |  |  |  |  |
| 17 | Depersonalization | -0.17** | -0.10** | -0.08* | -0.14** | -0.14** | -0.15** | -0.16** | 0.20** | 0.07* | 0.20** | 0.22** | 0.17** | -0.25** | 0.01 | 0.82** | 0.43** | 1 |  |  |  |  |
| 18 | Quality of Life | 0.28** | 0.04 | 0.20** | 0.20** | 0.20** | 0.31** | 0.33** | -0.17** | -0.15** | -0.17** | -0.29** | -0.15** | 0.27** | 0.17** | -0.40** | -0.42** | -0.24** | 1 |  |  |  |
| 19 | General Health | 0.26** | 0.04 | 0.16** | 0.14** | 0.20** | 0.33** | 0.34** | -0.18** | -0.20** | -0.15** | -0.28** | -0.16** | 0.28** | 0.21** | -0.34** | -0.38** | -0.18** | 0.60** | 1 |  |  |
| 20 | Presenteeism | -0.23** | -0.03 | -0.20** | -0.12** | -0.16** | -0.26** | -0.23** | 0.22** | 0.16** | 0.14** | 0.23** | 0.23** | -0.12** | -0.13** | 0.21** | 0.27** | 0.08* | -0.20** | -0.31** | 1 |  |
| 21 | Productivity | 0.22** | 0.22** | 0.10** | 0.17** | 0.18** | 0.12** | 0.21** | 0.01 | -0.06 | -0.07* | -0.03 | -0.04 | 0.34** | 0.06 | -0.30** | -0.23** | -0.29** | 0.29** | 0.23** | -0.05 | 1 |

Note. ** Correlation is significant at the p< 0.01 level (2-tailed).

* Correlation is significant at the p< 0.05 level (2-tailed).
